# Microbiological Findings and Risk Profiles in Hepatobiliary and Pancreatic Surgery Associated Surgical Site Infections: A Retrospective Cohort Study

**DOI:** 10.3390/pathogens14121215

**Published:** 2025-11-29

**Authors:** Mehmet Erinmez, Hatice Birgin, Latif Yılmaz, Yasemin Zer

**Affiliations:** 1 Department of Medical Microbiology, Faculty of Medicine, Gaziantep University, Gaziantep 27310, Türkiye; 2 Department of General Surgery, Faculty of Medicine, Gaziantep University, Gaziantep 27310, Türkiye

**Keywords:** surgical site infections, hepatobiliary surgery, pancreatic surgery, predictive risk model, microbiological profile, prolonged operative time, *Escherichia coli*

## Abstract

Surgical site infections (SSIs) are among the most frequent healthcare-associated infections, leading to prolonged hospitalization, increased costs, and impaired recovery. This retrospective cohort study aimed to identify the determinants and microbial patterns of SSIs following hepatobiliary and pancreatic (HPB) surgery to inform preventive strategies and optimize clinical outcomes. The patients undergoing hepatobiliary and pancreatic surgery from 2014 to 2024 in a tertiary university hospital are reviewed. SSI was defined according to Centers for Disease Control and Prevention (CDC) criteria, and microbiological isolates were identified through routine culture methods and susceptibility testing. Clinical, operative, and microbiological data of patients who underwent hepatobiliary and pancreatic surgery were extracted, including demographics, comorbidities, operative characteristics, and postoperative outcomes. Among 553 hepatobiliary and pancreatic surgery patients, SSI occurred in 48.6%. Gram-negative bacteria predominated, with *E. coli* as the leading pathogen. SSI was linked to open surgery, longer operative time, and higher ASA scores; malignancy, renal insufficiency, anemia, and COPD were independent risk factors. Age by itself was not a reliable predictor of infection, while operative duration demonstrated moderate predictive performance, with a sensitivity of 66%. These findings underscore the multifactorial pathogenesis of SSIs and emphasize the importance of refined perioperative strategies to mitigate postoperative infectious complications.

## 1. Introduction

Surgical site infections (SSIs) represent one of the most common healthcare-associated infections and a frequent complication of hospitalization, contributing to prolonged hospital stay, increased intensive care unit admissions, higher readmission rates, delays in adjuvant systemic therapy, and substantial costs [1,2]. In the United States alone, up to a quarter of a million cases of SSI are identified annually, reflecting a considerable burden in terms of reoperation, poor wound healing, cosmetic concerns, and impaired quality of life [3,4]. In low- and middle-income countries, the incidence of SSI is expected to be even higher, largely due to resource limitations, variability in infection control practices, and restricted access to standardized perioperative care [5].

The development of SSI involves multiple determinants and is related to patient-specific factors such as advanced age, comorbidities, obesity, malnutrition, immunosuppression, smoking, and malignancies, as well as surgical variables including wound contamination class, operative duration, and urgency of the procedure [6,7,8]. Emergency operations, in particular, carry a higher risk due to the frequent coexistence of these unfavorable conditions. While risk factors for SSI have been extensively studied in colorectal surgery and other gastrointestinal procedures, data on hepatobiliary and pancreatic resections remain limited [9,10]. HPB operative approaches include laparoscopic, robotic-assisted, endoscopic, and open surgery, with liver resections, pancreatic resections, and cholecystectomies being most commonly performed [11]. While minimally invasive techniques are increasingly adopted in resource-rich settings, open surgery remains the predominant approach, particularly for pancreaticoduodenectomy [12]. Pancreaticoduodenectomy involves pancreatojejunostomy, choledochojejunostomy, and gastrojejunostomy, procedures that often require prolonged operative time and are associated with an increased risk of bacterial contamination [13].

Hepatobiliary and pancreatic (HPB) surgery encompasses complex procedures involving the liver, pancreas, gallbladder, and bile ducts. Bile plays a crucial role in maintaining intestinal homeostasis by inhibiting bacterial overgrowth through its detergent properties and supporting the intestinal mucosa, which is the primary source of secretory IgA [14]. Although bile is typically sterile, bacterial colonization can occur in the presence of gallstones or biliary obstruction [15]. Bile leakage following liver resection and pancreatic fistula formation after pancreatic resection are frequent contributors to organ/space SSIs in patients undergoing HPB surgery [16]. Compared with other gastrointestinal operations, HPB procedures are associated with a disproportionately higher incidence of SSI, ranging between 9.9 and 23% in large cohort studies and national databases [13,17,18]. However, the incidence of SSIs following pancreaticoduodenectomy or hepatectomy with biliary reconstruction is notably high, exceeding 40% [19]. The increased risk can be attributed to the technical complexity of these operations and the potential exposure to contaminated bile and pancreatic secretions [20].

Post-operative infectious complications represent the leading cause of morbidity in HPB surgery, with SSIs accounting for a large proportion [21]. Most infections in patients with preoperative hospitalization were caused by microorganisms resistant to standard surgical prophylaxis, including methicillin-resistant *Staphylococcus aureus* (MRSA), vancomycin-resistant *Enterococcus* (VRE), and other multidrug-resistant bacteria [21,22]. Antimicrobial-resistant bacterial infection and colonization, especially after surgery, are a major global clinical problem that is expected to continue to increase in parallel with the increase in the elderly population [10]. Therefore, this study aimed to identify risk factors and characterize the microbial spectrum of surgical site infections after hepatobiliary and pancreatic surgery to guide clinical decision making.

## 2. Materials and Methods

This retrospective cohort study was conducted at Gaziantep University Hospital, a tertiary referral center in southeastern Türkiye, and included all patients who underwent hepatobiliary and pancreatic surgery between January 2014 and December 2024. All adult patients (≥18 years) who underwent eligible procedures, including liver resections, pancreatic resections (including pancreaticoduodenectomy), cholecystectomies, and biliary reconstructive surgeries, and who had complete clinical, operative, and postoperative follow-up data were included. SSI was defined according to the Centers for Disease Control and Prevention/NHSN criteria as infections occurring within 30 days of surgery, involving the incision or the operative organ space. Diagnosis required evidence of purulent drainage, positive culture from the surgical site, imaging or operative findings consistent with infection, incision dehiscence with clinical signs of inflammation, or a clinical diagnosis of SSI made by the treating surgeon [23]. All SSI events were identified from a prospectively maintained institutional database, based on surveillance conducted by the institutional infection control team. Comprehensive clinical and laboratory data of patients who underwent hepatobiliary and pancreatic surgery were extracted, including demographics, comorbidities, operative characteristics, and postoperative outcomes. The Institutional Review Board of Gaziantep University School of Medicine approved this study (decision no: 2025/219, and date of 6 August 2025). This study is reported in accordance with the STROBE guidelines for cohort studies. The following parameters were systematically collected and analyzed:

- Clinical data: Age, sex, ICU admission, primary diagnosis, affected organ, presence of malignancy, comorbidities (hypertension, diabetes mellitus, cardiac disease, renal failure, chronic obstructive pulmonary disease, liver disease), obesity (Body Mass Index (BMI) ≥ 30 kg/m^2^), anemia (hemoglobin (Hb) levels < 12.0 g/dL in women and <13.0 g/dL in men), albumin requirement, transfusion history, smoking status, infective microorganisms, and laboratory parameters (white blood cell count, hemoglobin).
- Operative variables: Type of operation, emergency versus elective setting, duration of surgery, length of preoperative hospitalization, need for reoperation and interval to reoperation and ASA score.
- Outcomes: Mortality (yes/no, time to mortality in days), postoperative complications.
- Exclusion criteria: Patients with a history of organ transplantation, those who underwent multiple hepatobiliary or pancreatic surgeries, and patients who died before postoperative day 30 without evidence of SSI were excluded because they did not meet the CDC 30-day criterion, and early mortality could bias the outcome assessment.

### 2.1. Microbiological Methods

Specimens (pus or wound swabs) were obtained from patients with signs of infection and processed by standard microbiological techniques. Cultures were inoculated onto Columbia agar with 5% sheep blood, chocolate agar, and EMB agar, and incubated at 36 ± 2 °C with 5% CO_2_ for 24–48 h. Gram staining and microscopic evaluation were performed to distinguish true pathogens from colonizers. This was achieved using the Q score system, in which polymorphonuclear cells (PMNs) indicate infection or inflammation and are assigned positive values, whereas squamous epithelial cells (SECs) suggest superficial contamination and are assigned negative values [24]. Identification of isolates was carried out using MALDI-TOF (Bruker, Karlsruhe, Germany). Antimicrobial susceptibility testing was performed using VITEK 2 (bioMérieux, Marcy l’Étoile, France), disc diffusion, or E-test, in accordance with EUCAST 2025 guidelines [25]. Anaerobic cultures were not performed; therefore, anaerobic bacteria are not represented in the reported microbial spectrum.

### 2.2. Statistical Analysis

Statistical analyses were performed using the SPSS software package (Statistical Package for the Social Sciences for Windows, Version 29.0; IBM Corp., Armonk, NY, USA). Descriptive statistics were presented as mean ± standard deviation for continuous variables and as frequency (n) and percentage (%) for categorical variables. Given the large sample size, parametric testing was deemed appropriate. Normality was assessed with the Kolmogorov–Smirnov test, and continuous variables were compared using the independent sample *t*-test. Comparisons of categorical variables were conducted using the Chi-square (χ^2^) test. To identify independent predictors associated with the development of surgical site infection, logistic regression analysis was performed. The relative risks of significant variables were expressed as Odds Ratios (OR) with 95% confidence intervals (CI). The explanatory power of the model was evaluated using the Nagelkerke R^2^ value. Additionally, ROC (Receiver Operating Characteristic) analysis was conducted to assess the discriminatory ability parameters in predicting surgical site infection. For each variable, cut-off values, sensitivity, specificity, and area under the curve (AUC) values were calculated. A *p*-value of <0.05 was considered statistically significant for all analyses.

## 3. Results

### 3.1. Patient Characteristics

A total of 553 patients who underwent hepatobiliary and pancreatic surgery were included in the study, and their demographic and clinical characteristics were evaluated. The mean age of the patients was 55.77 ± 16.98 years. The sex distribution was 48.5% female and 51.5% male. The incidence of SSI was 48.6%. Based on microbiological analyses, among the cases with positive wound cultures, Gram-negative bacteria were detected in 84.4%, Gram-positive bacteria in 13.8%, and fungal growth in 1.8% of isolates. These organisms are considered true pathogens based on clinical correlation and Gram-stain evaluation using the Q score system to differentiate infection from superficial contamination. However, it should be noted that not all SSIs yield positive cultures (17.2%) and that anaerobic bacteria do not grow in routine culture conditions. In our cohort, 59.5% of surgical site infections were polymicrobial. For polymicrobial infections, each isolated organism was evaluated individually and included in the microbiological analysis. *Escherichia coli* (44.6%) was the most frequently isolated pathogen, followed by *Klebsiella pneumoniae* (19.3%), *Pseudomonas aeruginosa* (10.8%), and *Enterococcus faecium* (9.3%). Multidrug-resistant (acquired resistance to at least one agent in three or more antimicrobial groups) isolates accounted for 56.7% of all recovered microorganisms, and detailed resistance rates of most common bacterial agents were presented in Table 1.

Regarding diagnostic distribution, cholelithiasis was the most common indication for surgery (42.1%), followed by hepatic pathologies (27.3%) and pancreatic diseases (19.2%). In terms of operative characteristics, 70.9% of the procedures were performed electively, whereas 29.1% were performed under emergency conditions. Laparoscopic surgery was utilized in 31.1% of cases, representing a markedly lower rate compared with open procedures. Overall, the findings indicate that this patient cohort primarily consisted of middle-aged individuals with a balanced sex distribution. Despite the high proportion of elective and laparoscopic interventions, SSI rates remained considerable. Additionally, Gram-negative bacteria predominated among the isolated microorganisms.

### 3.2. Risk Factor Evaluation for Operative Characteristics

When the relationship between SSI development and demographic or clinical variables was evaluated, no significant differences were observed between the groups in terms of age (*p* = 0.308) and sex (*p* = 0.210) (Table 2). Regarding operative characteristics, SSI rates were found to be significantly higher in patients who underwent open surgery compared with those treated with laparoscopic procedures (*p* = 0.001). However, whether the procedure was performed under emergency or elective conditions did not result in a significant difference in infection rates (*p* = 0.237).

Assessment of anesthesia risk classification revealed that patients categorized as ASA III had a significantly higher rate of infection (*p* = 0.001). In the evaluation of preoperative parameters, mean length of hospital stay was significantly longer in patients who developed SSI (*p* = 0.001). Similarly, the duration of surgery was significantly prolonged in the infection group compared with patients without infection (*p* = 0.001). A notable difference was also observed in the need for reoperation (*p* = 0.001), with both the frequency and interval to reoperation (*p* = 0.001) being higher among those who developed SSI. There was no significant difference between the groups in terms of mortality (*p* = 0.085), and the mortality rate among patients with SSI remained low (Table 2).

### 3.3. Risk Factor Evaluation for Comorbidities

The relationship between SSI development and metabolic or comorbidity-related variables was evaluated, revealing that several clinical factors were significantly associated with infection (Table 3). No differences were observed between the groups in terms of intensive care unit (ICU) admission (*p* = 0.743). In contrast, the presence of malignancy was strongly associated with SSI, with the infection rate rising to 34.6% (*p* = 0.001). When cardiometabolic comorbidities were assessed, the presence of hypertension (*p* = 0.001) and diabetes mellitus (*p* = 0.001) was found to significantly increase the risk of infection. Preoperative anemia demonstrated one of the strongest associations with SSI, with 57.2% of anemic patients developing postoperative infection (*p* = 0.001) (Table 3).

### 3.4. Predictive Performance of Risk Variables for SSI

According to the logistic regression analysis, potential determinants of surgical site infection (SSI) were first evaluated through univariate analysis, and variables found to be significant were subsequently included in a multivariate model (Table 4). The presence of malignancy, renal insufficiency, preoperative anemia, and COPD remained significantly associated with SSI development in both univariate and multivariate analyses (Table 4). In contrast, hypertension, diabetes mellitus, heart failure, transfusion requirement, and WBC count did not demonstrate a significant association with SSI occurrence (Table 4).

### 3.5. Discriminative Performance of SSI Risk Indicators

The discriminative performance of age, operative duration, and WBC level in predicting the development of surgical site infection was evaluated using ROC (Receiver Operating Characteristic) analysis at both univariate and multivariate levels. Age alone did not demonstrate adequate discriminative ability for predicting infection, whereas operative duration showed a sensitivity of 66% (Table 5). In the combined model including both age and operative duration, the sensitivity was 60%, specificity was 60%, and the AUC was 0.60 (Table 5).

## 4. Discussion

HPB surgeries are intricate operations with a substantial risk of SSI and are predominantly performed in individuals with malignant disease [26]. Prolonged surgery, heavy intraoperative bleeding, and procedural complexity have been recognized as key contributors to SSI following HPB operations [26]. In our study, longer operation time was significantly associated with SSI occurrence, confirming these observations and underscoring the importance of minimizing operative duration to reduce infection risk. Surgical approach emerged as another determinant of SSI in our cohort. Consistent with previous studies, patients who underwent open surgery demonstrated significantly higher rates of SSI compared to those who received minimally invasive procedures [27,28]. The elevated SSI incidence observed in open procedures may result from the larger incision area, higher risk of intraoperative contamination, and greater tissue trauma, all of which impair local perfusion and hinder wound healing [28]. Interestingly, there was no association with SSI and surgery type, meaning elective vs. emergency. However, in our findings, the need for reoperation and delayed requirement for reoperation had a statistically significant association with SSI development in HPB surgery.

Ejaz et al. [29] likewise found that blood transfusions elevate the risk of SSI and overall morbidity following HPB procedures, likely as a result of transfusion-related immunomodulatory effects. Consistent with the literature, intraoperative blood transfusion was associated with higher rates of SSI in our cohort. In a similar way, the requirement for albumin replacement was significantly associated with SSI. Previous hospitalization has been identified as a potential risk factor for SSI due to colonization with multidrug-resistant organisms [21]. In our study, this variable showed a statistically significant association, suggesting that the effectiveness of preoperative decolonization and antibiotic prophylaxis protocols warrants reassessment. In contrast, postoperative ICU stays over 48 h were not a significant contributor to SSI development. Unlike colorectal operations, where gut flora–related contamination is the main concern, HPB surgeries are particularly susceptible to bile and pancreatic leaks that promote organ/space infections [30,31]. The predominant pathogens identified in our study were *E. coli*, *Enterococcus* spp., and *Klebsiella* spp., aligning with the bile-derived microbial flora commonly associated with HPB surgical infections [26]. This distribution underscores the importance of tailoring perioperative antimicrobial prophylaxis to ensure adequate coverage against enteric Gram-negative bacteria and *Enterococcus* species.

Among patient-related factors, sex was not significantly associated with SSI in our cohort, which does not align with multiple prior studies discovering higher rates in males [32,33,34]. Aghdassi et al. [35] suggested that variations in bacterial skin colonization between sexes may partly account for these observed differences. However, our findings emphasize the need for further clarification about responsible biological mechanisms. Regarding age, our data indicated that there was no statistically significant association between advanced age and SSI. This finding partially aligns with Mentor et al. [33], who reported no such association, while contradicting Pędziwiatr et al. [36], who identified age as a risk factor in pancreatic surgery. The variability among studies may reflect differences in age cutoffs and surgical complexity. Nevertheless, the higher burden of comorbidities such as diabetes and cardiovascular disease among elderly patients likely contributes to impaired wound healing and an increased risk of infection [26,30,31,32,33]. Also, in our cohort, comorbidities such as malignancy, hypertension, diabetes, heart failure, renal failure, liver failure and COPD were significantly associated with SSI. Also, higher ASA scores and smoking were significantly associated with higher rates of SSI. In contrast, obesity had a limited association with SSI in our analysis, despite prior literature suggesting potential relevance [20,37]. This finding may indicate that enhanced perioperative metabolic and nutritional optimization has mitigated the influence of obesity on infection risk. Preoperative WBC levels also differed significantly between the SSI and non-SSI groups in our study. This observation supports the recognized utility of WBC count and other biomarkers (such as C-reactive Protein (CRP) and Procalcitonin (PCT)) as indicators of postoperative infectious complications in HPB surgery. Also, preoperative anemia was found as an independent and significant contributor to SSI in our study, which may be expected to be related to intraoperative blood transfusion. To our knowledge, this parameter has received limited consideration in studies examining SSI risk factors in HPB surgery.

Large multicenter studies report overall SSI rates ranging from 15.6% to 29.8%, with higher incidence observed after complex procedures such as hepatectomy with biliary reconstruction and pancreatectomy [9,13,26,32]. Meta-analytic data indicate SSI rates of 25.1% for pancreatic and 10.4% for liver resections, with organ/space SSI reaching 47.7% in patients with postoperative pancreatic fistula [33]. Among patients who experienced bile leak, the organ/space SSI rate reached 43% [13]. In our cohort, the overall SSI rate was markedly higher at 48.6%. This high overall rate is likely influenced by the fact that biliary system surgeries accounted for the majority of operations in our cohort at 51.3%. Our findings underscore the critical impact of surgery extent with multiorgan procedures exhibiting the greatest risk (83.9%), followed by biliary (51.1%), pancreatic (45.3%), and liver-only surgeries (37.9%) (*p* = 0.001). These differences likely reflect not only procedural complexity but also patient-related factors, including comorbidities such as diabetes, renal insufficiency, or immunosuppression, and nutritional status, all of which are established predictors of SSI. Furthermore, variations in healthcare infrastructure, perioperative and intraoperative resources between high-income and low-income settings may influence infection rates. This study has several limitations. The retrospective cohort design is a major limitation, and it was conducted at a single center, which may limit generalizability. Microbiological analyses were limited to routine aerobic cultures; anaerobic cultures and systematic bile or pancreatic juice cultures were not performed, potentially restricting insight into the full spectrum of pathogens and resistance patterns. Antimicrobial prophylaxis regimens and previous antibiotic exposure were not analyzed in detail, and advanced molecular diagnostics were not employed, which may have led to underdetection of certain fastidious or multidrug-resistant organisms. The study covers an 11-year period, during which clinical practices and laboratory protocols may have evolved. However, despite minor changes in preoperative, intraoperative, and postoperative management protocols, the large number of patients included each year ensured representative and robust data for the entire cohort.

## 5. Conclusions

In conclusion, this study identified multiple significant determinants of surgical site infection in HPB surgery. Malignancy, renal insufficiency, preoperative anemia, and COPD were found to be independent predictors of SSI after HPB surgery. The results highlight the multifactorial etiology of SSIs and the need for optimized perioperative strategies, including reduced operative duration, prudent transfusion and albumin use, and targeted antimicrobial prophylaxis, to minimize postoperative infection risk.

## Figures and Tables

**Table 1:** Antimicrobial resistance rates of common clinical isolates.

Antimicrobial Agent	*E. coli*	*K. pneumoniae*	*P. aeruginosa*	Antimicrobial Agent	*Enterococcus* spp.	*S. aureus*
Ampicillin	51.6%	NT	NT	Ampicillin	8.3%	35.2%
Amoxicillin Clavulanate	43.5%	58.8%	NT	Amoxicillin Clavulanate	5.5%	NT
Piperacillin-Tazobactam	54.0%	71.7%	48.5%	Oxacillin	NT	41.1%
Cefuroxime	51.5%	56.9%	NT	Benzylpenicillin	5.5%	94.1%
Cefoxitin	NT	16.2%	NT	Gentamicin	8.3%	NT
Ceftriaxone	61.6%	65.0%	NT	Tobramycin	NT	47.0%
Ceftazidime	52.3%	66.9%	10.5%	Streptomycin	16.6%	NT
Cefepime	60.0%	63.2%	50.0%	Ciprofloxacin	NT	29.4%
Ceftaroline	23.8%	18.6%	NT	Levofloxacin	NT	29.4%
Imipenem	6.1%	18.6%	11.9%	Moxifloxacin	NT	5.8%
Meropenem	15.4%	50.7%	39.4%	Tetracycline	NT	64.7%
Ertapenem	32.2%	60.7%	NT	Tigecycline	0%	11.7%
Amikacin	2.0%	21.0%	13.3%	Vancomycin	0%	0%
Gentamicin	30.8%	47.8%	21.1%	Teicoplanin	0%	0%
Tobramycin	NT	NT	6.3%	Linezolid	0%	5.8%
Netilmicin	25.5%	39.2%	28.8%	Daptomycin	0%	5.8%
Ciprofloxacin	65.5%	75.5%	39.4%	Trimethoprim-Sulfamethoxazole	NT	0%
Tigecycline	0.6%	29.6%	NT	Erythromycin	NT	47.0%
Trimethoprim-Sulfamethoxazole	58.1%	65.0%	NT	Clindamycin	NT	47.0%
Colistin	1.0%	5.7%	2.8%	Fusidic Acid	NT	23.5%
				Rifampicin	NT	58.8%

NT: Not tested.

**Table 2:** Demographic and operative characteristics evaluation for SSI development.

Parameters	Surgical Site Infection		*p* Value
	Present	Absent	
Age ($\bar{x}$ ± sd)	56.1 ± 16.4	55.4 ± 17.4	^1^ 0.308
Sex (n,%)			
Female	123 (45.7%)	145 (51.1%)	^2^ 0.210
Male	146 (54.3%)	139 (48.9%)	
Surgery type			
Emergency	72 (26.8%)	89 (32.3%)	^2^ 0.237
Elective	197 (73.2%)	295 (68.7%)	
Surgery technique			
Open	206 (76.6%)	175 (61.6%)	^2^ 0.001 *
Laparoscopic	63 (23.4%)	109 (38.4%)	
Extent of surgery (n,%)			
Biliary system	145 (53.9%)	139 (48.9%)	^2^ 0.001 *
Liver	50 (18.6%)	82 (28.9%)	
Pancreas	48 (17.8%)	58 (20.4%)	
Multiorgan	26 (9.7%)	5 (1.8%)	
ASA			
ASA I	23 (8.6%)	43 (15.1%)	^2^ 0.001 *
ASA II	77 (28.6%)	121 (42.6%)	
ASA III	169 (62.8%)	120 (42.3%)	
Preoperative hospital stay (days; $\bar{x}$ ± sd)	4 ± 5.7	2.4 ± 4.4	^1^ 0.001 *
Duration of surgery (minutes; $\bar{x}$ ± sd)	180.4 ± 130.7	140 ± 134.3	^1^ 0.001 *
Requirement for re-operation (n,%)			
Yes	77 (28.6%)	40 (14.1%)	^2^ 0.001 *
No	192 (71.4%)	244 (85.9%)	
Time to reoperation (days; $\bar{x}$ ± sd)	3.6 ± 7.6	1.7 ± 6	^1^ 0.001 *
90-day-Mortality ($\bar{x}$ ± sd)	0.08 ± 0.28	1.43 ± 6.26	^1^ 0.309
Mortality (days; $\bar{x}$ ± sd)	2.33 ± 9.01	11.72 ± 5.24	^1^ 0.085

^1^ Independent Sample *t* Test, ^2^ Chi-Square Analysis, * *p* < 0.05.

**Table 3:** Evaluation of metabolic and comorbidity risk factors for SSI.

Parameters	Surgical Site Infection		*p* Value
	Present	Absent	
Malignancy (n,%)			
Present	93 (34.6%)	43 (15.1%)	^2^ 0.001 *
Absent	176 (65.4%)	241 (84.9%)	
Hypertension (n,%)			
Present	92 (34.2%)	60 (21.1%)	^2^ 0.001 *
Absent	177 (65.8%)	224 (78.9%)	
Diabetes mellitus (n,%)			
Present	93 (34.6%)	60 (21.1%)	^2^ 0.001 *
Absent	176 (65.4%)	224 (78.9%)	
Heart failure (n,%)			
Present	70 (26.0%)	18 (6.3%)	^2^ 0.001 *
Absent	199 (74.0%)	266 (93.7%)	
Renal Failure (n,%)			
Present	37 (13.8%)	5 (1.8%)	^2^ 0.001 *
Absent	232 (86.2%)	279 (98.2%)	
Liver Failure (n,%)			
Present	13 (4.8%)	1(0.4%)	^2^ 0.001 *
Absent	256 (95.2%)	283 (99.6%)	
Chronic Obstructive Pulmonary Disease (n,%)			
Present	24 (8.9%)	2 (0.7%)	^2^ 0.001 *
Absent	245 (91.1%)	282 (99.3%)	
Obesity (n,%)			
Present	43 (16.0%)	29 (10.2%)	^2^ 0.044 *
Absent	226 (84.0%)	255 (89.8%)	
Preoperative anemia (n,%)			
Present	154 (57.2%)	88 (31.0%)	^2^ 0.001 *
Absent	115 (42.8%)	196 (69.0%)	
Preoperative WBC (10^9^/L; $\bar{x}$ ± sd)	12.88 ± 6.02	11.72 ± 5.24	^1^ 0.008 *
Smoking (n,%)			
Present	101 (37.5%)	41 (14.4%)	^2^ 0.001 *
Absent	168 (62.5%)	243 (85.6%)	
Requirement for Albumin (n,%)			
Present	99 (36.8%)	68 (23.9%)	^2^ 0.001 *
Absent	170 (63.2%)	216 (76.1%)	
Requirement for Transfusion (n,%)			
Present	54 (20.1%)	34 (12.0%)	^2^ 0.009 *
Absent	215 (79.9%)	250 (88.0%)	
Postoperative ICU stay over 48 h (n,%)			
Present	39 (14.5%)	44 (15.5%)	^2^ 0.743
Absent	230 (85.5%)	240 (84.5%)	

^1^ Independent Sample *t* Test, ^2^ Chi-Square Analysis, * *p* < 0.05.

**Table 4:** Logistic regression analysis for determining factors associated with SSI.

Parameters	Surgical Site Infection			
	Univariate		Multivariate	
	OR (%95 CI)	*p* Value	OR (%95 CI)	*p* Value
Age	0.98 (0.97–1.00)	0.026 *	0.99 (0.98–1.00)	0.175
Sex				
Male	1	-		
Female	0.95 (0.64–1.40)	0.807		
ASA				
ASA I	1	-		
ASA II	1.06 (0.56–2.02)	0.843		
ASA III	1.73 (0.85–3.53)	0.130		
Surgery type				
Emergency	1	-		
Elective	1.13 (0.71–1.79)	0.601		
Surgery technique				
Laparoscopic	1	-		
Open	1.58 (0.95–2.61)	0.075		
Duration of surgery	1.00 (0.99–1.00)	0.269		
Postoperative ICU stay				
Absent	1	-		
Present	0.77 (0.44–1.32)	0.347		
Malignancy				
Absent	1	-	1	-
Present	1.93 (1.14–3.23)	0.013 *	2.49 (1.60–3.89)	0.001 *
Hypertension				
Absent	1	-		
Present	1.22 (0.74–2.01)	0.421		
Diabetes mellitus				
Absent	1	-		
Present	0.85 (0.51–1.39)	0.521		
Renal failure				
Absent	1	-	1	-
Present	5.41 (1.83–15.94)	0.002 *	6.49 (2.41–17.47)	0.001 *
COPD				
Absent	1	-	1	-
Present	10.95 (2.36–50.79)	0.002 *	11.89 (2.63–53.64)	0.001 *
Heart failure				
Absent	1	-		
Present	7.76 (0.83–71.80)	0.071		
Preoperative anemia				
Absent	1	-	1	-
Present	2.58 (1.68–3.97)	0.001 *	2.47 (1.70–3.58)	0.001 *
Requirement for transfusion				
Absent	1	-		
Present	0.84 (0.46–1.52)	0.573		
WBC	1.02 (0.99–1.06)	0.142		

Logistic Regression (R^2^: 0.26); * *p* < 0.05.

**Table 5:** ROC analysis of association between risk factors and SSI.

Parameter	Cut-Off Value	Sensitivity (%)	Specificity (%)	AUC (%95 CL)	*p* Value
Age	56.5	0.55	0.50	0.51	0.728
Duration of surgery (min)	117.5	0.66	0.54	0.56	0.019 *
WBC (10^9^/L)	11.45	0.54	0.60	0.58	0.020 *
Age * Surgery duration	-	0.60	0.60	0.60	0.000 *
Age * WBC	-	0.55	0.55	0.55	0.045 *
Surgery duration * WBC	-	0.59	0.41	0.63	0.000 *
Age * Surgery duration * WBC	-	0.58	0.42	0.61	0.000 *

ROC Analysis; * *p* < 0.05

## Data Availability

The data that support the findings of this study are available from the corresponding author upon reasonable request.

## Abbreviations

The following abbreviations are used in this manuscript:

SSI	Surgical site infection
HPB	Hepatobiliary and pancreatic
ICU	Intensive care unit
BMI	Body-Mass Index

## References

[1] Al-Dabbagh M.A., Dobson S. (2013). The evidence behind prophylaxis and treatment of wound infection after surgery. Adv. Exp. Med. Biol..

[2] de Lissovoy G., Fraeman K., Hutchins V., Murphy D., Song D., Vaughn B.B. (2009). Surgical site infection: Incidence and impact on hospital utilization and treatment costs. Am. J. Infect. Control.

[3] Anderson D.J., Podgorny K., Berrios-Torres S.I., Bratzler D.W., Dellinger E.P., Greene L., Nyquist A.C., Saiman L., Yokoe D.S., Maragakis L.L. (2014). Strategies to prevent surgical site infections in acute care hospitals: 2014 update. Infect. Control. Hosp. Epidemiol..

[4] Magill S.S., Edwards J.R., Bamberg W., Beldavs Z.G., Dumyati G., Kainer M.A., Lynfield R., Maloney M., McAllister-Hollod L., Nadle J. (2014). Multistate point prevalence survey of health care-associated infections. N. Engl. J. Med..

[5] (2018). Global Guidelines for the Prevention of Surgical Site Infection.

[6] Imamura K., Adachi K., Sasaki R., Monma S., Shioiri S., Seyama Y., Miura M., Morikawa Y., Kaneko T. (2016). Randomized Comparison of Subcuticular Sutures Versus Staples for Skin Closure After Open Abdominal Surgery: A Multicenter Open-Label Randomized Controlled Trial. J. Gastrointest. Surg..

[7] Tsujinaka T., Yamamoto K., Fujita J., Endo S., Kawada J., Nakahira S., Shimokawa T., Kobayashi S. (2013). Subcuticular sutures versus staples for skin closure after open gastrointestinal surgery: A phase 3, multicentre, open-label, randomised controlled trial. Lancet.

[8] De Simone B., Sartelli M., Coccolini F., Ball C.G., Brambillasca P., Chiarugi M., Campanile F.C., Nita G., Corbella D., Leppaniemi A. (2020). Intraoperative surgical site infection control and prevention: A position paper and future addendum to WSES intra-abdominal infections guidelines. World J. Emerg. Surg..

[9] Morikane K., Honda H., Yamagishi T., Suzuki S., Aminaka M. (2014). Factors associated with surgical site infection in colorectal surgery: The Japan nosocomial infections surveillance. Infect. Control Hosp. Epidemiol..

[10] Niitsuma T., Kusachi S., Takesue Y., Mikamo H., Asai K., Watanabe M. (2019). Current status of postoperative infections after digestive surgery in Japan: The Japan Postoperative Infectious Complications Survey in 2015. Ann. Gastroenterol. Surg..

[11] Bismuth H., Majno P.E. (2000). Hepatobiliary surgery. J. Hepatol..

[12] Cawich S.O., Kluger M.D., Francis W., Deshpande R.R., Mohammed F., Bonadie K.O., Thomas D.A., Pearce N.W., Schrope B.A. (2021). Review of minimally invasive pancreas surgery and opinion on its incorporation into low volume and resource poor centres. World J. Gastrointest. Surg..

[13] Nakahira S., Shimizu J., Miyamoto A., Kobayashi S., Umeshita K., Ito T., Monden M., Doki Y., Mori M. (2013). Proposal for a sub-classification of hepato-biliary-pancreatic operations for surgical site infection surveillance following assessment of results of prospective multicenter data. J. Hepatobiliary Pancreat. Sci..

[14] Isik O., Kaya E., Sarkut P., Dundar H.Z. (2015). Factors Affecting Surgical Site Infection Rates in Hepatobiliary Surgery. Surg. Infect..

[15] Mohamed A.H., Mohamud H.A., Arslan E. (2021). Epidemiological Characteristics and Predisposing Factors for Surgical Site Infections Caused by Bacterial Pathogens Exhibiting Multidrug-Resistant Patterns. Antibiotics.

[16] Sugiura T., Uesaka K., Ohmagari N., Kanemoto H., Mizuno T. (2012). Risk factor of surgical site infection after pancreaticoduodenectomy. World J. Surg..

[17] Edwards J.R., Peterson K.D., Mu Y., Banerjee S., Allen-Bridson K., Morrell G., Dudeck M.A., Pollock D.A., Horan T.C. (2009). National Healthcare Safety Network (NHSN) report, data summary for 2006 through 2008, issued December 2009. Am. J. Infect. Control.

[18] Yang T., Tu P.A., Zhang H., Lu J.H., Shen Y.N., Yuan S.X., Lau W.Y., Lai E.C., Lu C.D., Wu M.C. (2014). Risk factors of surgical site infection after hepatic resection. Infect. Control Hosp. Epidemiol..

[19] Kimura F., Shimizu H., Yoshidome H., Ohtsuka M., Kato A., Yoshitomi H., Nozawa S., Furukawa K., Mitsuhashi N., Sawada S. (2006). Increased plasma levels of IL-6 and IL-8 are associated with surgical site infection after pancreaticoduodenectomy. Pancreas.

[20] Berríos-Torres S.I., Umscheid C.A., Bratzler D.W., Leas B., Stone E.C., Kelz R.R., Reinke C.E., Morgan S., Solomkin J.S., Mazuski J.E. (2017). Centers for Disease Control and Prevention Guideline for the Prevention of Surgical Site Infection, 2017. JAMA Surg..

[21] Dong Z.M., Chidi A.P., Goswami J., Han K., Simmons R.L., Rosengart M.R., Tsung A. (2015). Prior inpatient admission increases the risk of post-operative infection in hepatobiliary and pancreatic surgery. HPB.

[22] Siegel J.D., Rhinehart E., Jackson M., Chiarello L., Healthcare Infection Control Practices Advisory Committee (2007). Management of multidrug-resistant organisms in health care settings, 2006. Am. J. Infect. Control.

[23] Horan T.C., Andrus M., Dudeck M.A. (2008). CDC/NHSN surveillance definition of health care-associated infection and criteria for specific types of infections in the acute care setting. Am. J. Infect. Control.

[24] Matkoski C., Sharp S.E., Kiska D.L. (2006). Evaluation of the Q score and Q234 systems for cost-effective and clinically relevant interpretation of wound cultures. J. Clin. Microbiol..

[25] The European Committee on Antimicrobial Susceptibility Testing (2025). Breakpoint Tables for Interpretation of MICs and Zone Diameters. Version 15.0. https://www.eucast.org.

[26] Takahashi Y., Takesue Y., Fujiwara M., Tatsumi S., Ichiki K., Fujimoto J., Kimura T. (2018). Risk factors for surgical site infection after major hepatobiliary and pancreatic surgery. J. Infect. Chemother..

[27] Rosenthal R., Weber W.P., Marti W.R., Misteli H., Reck S., Dangel M., Oertli D., Widmer A.F. (2010). Surveillance of surgical site infections by surgeons: Biased underreporting or useful epidemiological data?. J. Hosp. Infect..

[28] Hassan R.S.E.E., Osman S.O.S., Aabdeen M.A.S., Mohamed W.E.A., Hassan R.S.E.E., Mohamed S.O.O. (2020). Incidence and root causes of surgical site infections after gastrointestinal surgery at a public teaching hospital in Sudan. Patient Saf. Surg..

[29] Ejaz A., Spolverato G., Kim Y., Margonis G.A., Gupta R., Amini N., Frank S.M., Pawlik T.M. (2015). Impact of blood transfusions and transfusion practices on long-term outcome following hepatopancreaticobiliary surgery. J. Gastrointest. Surg..

[30] Sadamori H., Yagi T., Shinoura S., Umeda Y., Yoshida R., Satoh D., Nobuoka D., Utsumi M., Yoshida K., Fujiwara T. (2013). Risk factors for organ/space surgical site infection after hepatectomy for hepatocellular carcinoma in 359 recent cases. J. Hepatobiliary Pancreat. Sci..

[31] Liu Q.Y., Zhang W.Z., Xia H.T., Leng J.J., Wan T., Liang B., Yang T., Dong J.H. (2014). Analysis of risk factors for postoperative pancreatic fistula following pancreaticoduodenectomy. World J. Gastroenterol..

[32] Chambers L.E., Sheen A.J., Whitehead K.A. (2022). A systematic review on the incidence and risk factors of surgical site infections following hepatopancreatobiliary (HPB) surgery. AIMS Bioeng..

[33] Mentor K., Ratnayake B., Akter N., Alessandri G., Sen G., French J.J., Manas D.M., Hammond J.S., Pandanaboyana S. (2020). Meta-Analysis and Meta-Regression of Risk Factors for Surgical Site Infections in Hepatic and Pancreatic Resection. World J. Surg..

[34] Mazmudar A., Vitello D., Chapman M., Tomlinson J.S., Bentrem D.J. (2017). Gender as a risk factor for adverse intraoperative and postoperative outcomes of elective pancreatectomy. J. Surg. Oncol..

[35] Aghdassi S.J.S., Schröder C., Gastmeier P. (2019). Gender-related risk factors for surgical site infections. Results from 10 years of surveillance in Germany. Antimicrob. Resist. Infect. Control.

[36] Pędziwiatr M., Małczak P., Mizera M., Witowski J., Torbicz G., Major P., Pisarska M., Wysocki M., Jankowski M., Rubinkiewicz M. (2018). Pancreatoduodenectomy for pancreatic head tumors in the elderly—Systematic review and meta-analysis. Surg. Oncol..

[37] Bone M., Latimer S., Walker R.M., Thalib L., Gillespie B.M. (2025). Risk factors for surgical site infections following hepatobiliary surgery: An umbrella review and meta-analyses. Eur. J. Surg. Oncol..

